# Hypertension in Northern Angola: prevalence, associated factors, awareness, treatment and control

**DOI:** 10.1186/1471-2458-13-90

**Published:** 2013-01-31

**Authors:** João E Pires, Yuri V Sebastião, António J Langa, Susana V Nery

**Affiliations:** 1 CISA Project (Health Research Center in Angola), Rua Direita do Caxito, Caxito, Bengo, Angola

**Keywords:** Angola, DSS, Hypertension, Prevalence, Epidemiological transition

## Abstract

**Background:**

Seventy-five million people are estimated to be hypertensive in sub-Saharan Africa. This translates in high morbidity and mortality, as hypertension is now considered to be the number one single risk factor for death worldwide. Accurate data from countries lacking national disease surveillance is needed to guide future evidence-driven health policies. The authors aimed to estimate the prevalence, awareness, management and control of hypertension and associated factors in an adult population of Angola.

**Methods:**

A community-based survey of 1,464 adults, following the World Health Organization's Stepwise Approach to Chronic Disease Risk Factor Surveillance, was conducted to estimate the prevalence of hypertension, awareness, treatment and control in Dande, Northern Angola. Using a demographic surveillance system database, a representative sample of subjects, stratified by sex and age (18–40 and 41–64 years old), was selected.

**Results:**

Prevalence of hypertension (systolic blood pressure ≥140 mmHg and/or diastolic blood pressure ≥90 mmHg and/or hypertensive therapy) was of 23% (95% CI: 21% to 25.2%). A follow-up consultation confirmed the hypertensive status in 82% of the subjects who had a second measurement on average 23 days after the first. Amongst hypertensive individuals, 21.6% (95% CI: 17.0% to 26.9%) were aware of their status. Only 13.9% (95% CI: 5.9% to 29.1%) of the subjects aware of their condition were under pharmacological treatment, of which approximately one-third were controlled. Older age, lower level of education, higher body mass index and abdominal obesity were found to be significantly (p<0.01) associated with hypertension.

**Conclusions:**

Our survey is the first to provide insightful data on hypertension prevalence in Angola. There is an urgent need for strategies to improve prevention, diagnosis and access to adequate treatment in this country, where a massive economic growth and consequent potential impact on lifestyle risk factors could lead to an increase in the prevalence of hypertension and cardiovascular disease.

## Background

In 2008, 17 million deaths occurred worldwide from cardiovascular diseases (CVDs), of which 80% occurred in middle and low-income countries [1]. Accounting for more than 7 million lives lost annually and for 57 million disability-adjusted life years, arterial hypertension is not only a major risk factor for CVDs, but also recognized as the number one single risk factor for death worldwide [2]. The majority of hypertensive individuals lives in developing regions, with recent estimates pointing to 75 million suffering from this condition in Sub-Saharan Africa (SSA), and a projected 125.5 million people affected by 2025 [3]. It is acknowledged, however, that limitations regarding the availability of data [1,4-6] surround predictive models such as those proposed by Twagirumuzika et al. [3]. The same authors stress the importance of using reproducible methodologies such as the World Health Organization's Stepwise Approach to Chronic Disease Risk Factor Surveillance (STEPS), in order to produce representative and accurate data [3,7]. When combined with Demographic Surveillance Systems (DSSs), this approach has been recognized as a powerful tool for generating information that can be used in guiding control and prevention of non-communicable diseases' (NCDs), as in this context, its longitudinal capability can detect the dynamics of NCDs and associated factors at the population level [8].

Located in southern Africa, Angola lacks national disease surveillance data on NCDs, more specifically on those associated with CVDs [1,9]. Despite having endured a 27-year conflict period that ended in 2002, Angola has been repeatedly ranked among the 3 fastest-growing economies in the World [10]. Given this economic growth and consequent impact on risk factors for CVDs such as hypertension and obesity, hypertension is likely to become an increasingly important public health problem in Angola.

In 2007 the CISA project (*Centre for Health Research in Angola*, translated) was established as a result of a partnership between the Angolan and Portuguese Governments and the Calouste Gulbenkian Foundation. CISA’s Demographic Surveillance System (DSS) monitors over 60,000 people, providing reliable demographic information and facilitating the implementation of epidemiological studies [11,12].

The aim of this study was to estimate the prevalence, awareness, management and control of hypertension and associated factors in an adult population of Angola through collection of baseline data utilizing the WHO STEPS' approach and our DSS as a platform. The public health implications of our findings are discussed in light of the current economic boom in Angola.

## Methods

### Study site and design

This community-based survey was conducted in Bengo Province, Northern Angola in CISA's DSS study area, which includes three communes (Caxito, Mabubas and Úcua) of the Dande municipality. Located 60 km north of Luanda, this area covers a population of 60,000 people, spread across 4,700 km^2^[11,12].

Following the WHO STEPS methodology [7], a representative sex- and age (18–40 and 41–64 years) stratified random sample was drawn from the DSS adult population database, which comprises 29,614 uniquely identified individuals aged 18–64 years old. Due to logistic constraints, probability proportionate to size sampling was used to choose 35 (out of 69) hamlets in the study area from which eligible adults could be selected. Fieldwork was conducted during a 12-week period, between October and December 2011, allowing for the recruitment of 1,464 of the selected subjects.

### Measurement of potential risk factors for hypertension

Information on sociodemographic characteristics and behavioural risk factors (i.e. alcohol and tobacco) was gathered using a questionnaire adapted from the WHO’s STEPS manual (version 2.1) [7], which was translated to Portuguese (official language) and Quimbundo (a national language), pre-tested and piloted before data collection. Frequent alcohol drinkers were defined as subjects that would consume alcohol 3 or more days per week and tobacco users were defined as subjects currently smoking tobacco on a daily basis. The questionnaire was expanded to include questions regarding hypertension related awareness (“ever measured blood pressure”, if yes, “ever told to be hypertensive”) and pharmacological or non-pharmacological management of hypertension (advices on diet change, weight loss, exercise or smoking). Place of residence (urban versus rural) was classified according to the definition provided by the National Institute of Statistics [13]. Information on level of education was obtained from the DSS database and categorized per number of years of schooling completed: none; one to four; five to eight and nine or more.

### Assessment of hypertension prevalence, awareness, treatment, control and follow-up

The following physical measurements were taken using standardized and internationally validated instruments: blood pressure, weight, height, waist and hip circumferences [14]. Blood pressure was measured using Omron M6 automatic sphygmomanometer (HEM-7211-E8 (V)) as recommended [15]. Three readings were taken after a 15-minute rest, three minutes apart. Measurements were performed with the participant seated, on the right arm and using the appropriate cuff size (22x32 cm or 32x42 cm). For data analysis, the average of the last two readings was used, as postulated by WHO [7]. Hypertension was defined as having a systolic blood pressure (SBP) of ≥140 mmHg and/or diastolic blood pressure (DBP) of ≥90 mmHg and/or reporting use of anti-hypertensive drug therapy in the previous two weeks. Pre-hypertension was defined as having SBP levels between 120 and 140 mmHg or DBP between 80 and 90 mmHg. Awareness was assessed by asking hypertensive subjects if they had ever been informed to be hypertensive by a health professional. Individuals taking anti-hypertensive medication at the time of survey administration were considered to be controlled if their blood pressure levels were below the thresholds aforementioned. Anthropometric measurements were taken with participants wearing light clothing and no shoes. Weight was measured to the nearest 0.1 Kg using a SECA 803 digital scale. Height was evaluated to the nearest 0.1 cm, using a SECA 213 portable stadiometer with the individual in the standing position. Waist and hip circumferences were measured to the nearest 0.1 cm using SECA 203 circumference tape following standard procedures [14]. Body mass index (BMI) was calculated as weight (Kg) divided by squared height (m^2^) and categorized as proposed by WHO [16]: <25 Kg/m^2^; 25–30 Kg/m^2^ (overweight) and ≥30 Kg/m^2^ (obese). Waist to hip ratio was calculated to assess abdominal obesity as defined by WHO: ≥0.9 for men and ≥0.85 for women [17]. Certified nurses conducted data collection after a one-week training-period. A follow-up consultation for the participants classified as hypertensive at the time of data collection was set up in collaboration with the Bengo General Hospital. In agreed consultation days, the attending cardiologist, undertook a single blood pressure measurement after 15 minutes of rest. This measurement was used to confirm the hypertensive diagnosis, if SBP above 140 mmHg and/or DBP above 90 mmHg. At the end of the follow-up period, a survey on perceived barriers to this hypertension follow-up was conducted among subjects who did not attend their scheduled appointment. A close-ended questionnaire was used for this purpose.

### Data management and statistics

Data was double entered into a PostgreSQL® database system. Analysis was conducted using STATA/SE v.11 software. Sociodemographic characteristics, anthropometric and physical measurements and hypertension status of participants were summarized using frequencies (percentages) or means and standard deviations. Taking advantage of the population sex and age (18–40 versus 41–64 years of age) strata distribution information available from our DSS, post stratification survey weights were calculated and used when estimating prevalence of hypertension and pre-hypertension, and when conducting all tests of significance. Significance level was set at p<0.05 for all hypothesis tests. Contingency tables with Pearson chi-squared (*χ*^2^) tests and pairwise correlations were used to identify factors associated with hypertension (yes/no, with subjects who had pre-hypertension grouped with no hypertension) and blood pressure (mean SBP and mean DSP), respectively. Pertinent variables with a significant *χ*^2^ statistic were used to build a multivariable logistic regression model for predicting hypertension status in women and men separately. The final model was evaluated using the area under the ROC curve and the F-adjusted mean residual goodness-of-fit test. Estimated prevalences and odds ratios (OR) are reported with corresponding 95% confidence intervals (95% CI).

### Ethics

The study protocol was approved by the Angolan Ministry of Health Ethics Committee and written informed consent was obtained from all the participants before data was collected.

## Results

### The study population

There were 1,464 participants in this survey, with the following distribution per strata: 611 women aged 18–40, 255 women aged 41–64; 449 men aged 18–40, 149 men aged 41–64. The mean age of the sample was 33.7 years old (SD 12.5), with women being slightly older than men on average (34.3 versus 32.7 years). The majority of the population was estimated to be living in an urban setting (87.9%; 95% CI: 86.1% to 89.4%) (Table 1). Just over one fifth never attended school and 17.9% (95% CI: 16.1% to 20.0%) had a high-school or higher level of education. Approximately half of the population drank alcohol frequently, with a higher proportion of frequent drinkers among men (58.8%; 95% CI: 54.9% to 62.7%) than among women (38.2%; 95% CI: 35.0% to 41.5%). Overall just over 10% of the population had active smoking habits, this percentage being lower in women (4.3%; 95% CI: 3.2% to 5.9%) compared to men (18.3%; 95% CI: 15.4% to 21.6%). The proportion of overweight and obese varied across gender, being lower in men (14.4% (95% CI: 11.8% to 17.5%) and 3.1% (95% CI: 2.0% to 4.9%) respectively) than in women (24.1 (95% CI: 21.2% to 27.3%) and 10.3% (95% CI: 8.4% to 12.7%) respectively). Forty percent (95% CI: 37.8% to 42.3%) of the population was estimated to have abdominal obesity (Table 1).

**Table 1:** Characteristics of the participants

**Characteristics**	**Female (n=866; 59.1%)**		**Male (n=598; 40.9%)**		**All participants (n=1464; 100.0%)**	
	**% Unweighted**	% Weighted* (95% CI)	**% Unweighted**	% Weighted* (95% CI)	**% Unweighted**	% Weighted* (95% CI)
**Place of residence**						
**Urban**	87.2	86.9 (84.6-89.0)	89.3	88.8 (86.1-91.1)	88.0	87.9 (86.1-89.4)
**Rural**	12.8	13.1 (11.0-15.4)	10.7	11.2 (8.9-13.9)	12.0	12.1 (10.6-13.9)
**Education**						
**None**	29.0	29.5 (26.7-32.4)	12.2	12.4 (10.0-15.3)	22.1	21.2 (19.3-23-2)
**1-4 years**	34.1	34.0 (30.9-37.2)	14.6	14.8 (12.2-17.9)	26.1	24.7 (22.6-26.9)
**5-8 years**	28.4	28.1 (25.3-31.1)	45.0	44.7 (40.8-48.7)	35.2	36.2 (33.8-28.9)
≥**9 years**	8.6	8.4 (6.8-10.4)	28.3	28.0 (24.6-31.8)	16.6	17.9 (16.1-20.0)
**Body Mass Index**						
**<25**	65.5	65.4 (62.2-68.5)	82.8	82.5 (79.2-85.3)	73.2	74.4 (72.0-76.6)
**25-30**	24.7	24.8 (22.0-27.8)	14.2	14.4 (11.8-17.5)	19.7	19.1 (17.1-21.3)
≥**30**	9.8	10.3 (8.0-12.0)	3.0	3.1 (2.0-4.9)	7.1	6.6 (5.4-8.0)
**Waist to hip ratio**						
**<0.85 women/<0.9 men**	42.6	42.4 (39.2-45-7)	79.1	78.5 (75.1-81.5)	59.7	60.0 (57.7-62.2)
**≥0.85 women/≥0.9 men**	57.4	57.6 (54.3-60.8)	20.9	21.5 (18.5-24.9)	40.3	40.0 (37.8-42.3)
**Alcohol consumption**						
**<3 d/wk**	61.7	61.8 (58.5-65.0)	41.3	41.2 (37.3-45.2)	53.4	51.8 (49.2-54.3)
≥**3 d/wk**	38.3	38.2 (35.0-41.5)	58.7	58.8 (54.9-62.7)	46.6	48.2 (45.7-50.8)
**Tobacco**						
**No**	95.8	95.7 (94.1-96.9)	82.1	81.7 (78.4-84.6)	90.2	88.9 (87.1-90.4)
**Yes**	4.2	4.3 (3.2-5.9)	17.9	18.3 (15.4-21.6)	9.8	11.1 (9.6-12.9)

* post-stratification weights used as described in the methods section.

**Figure 1:**
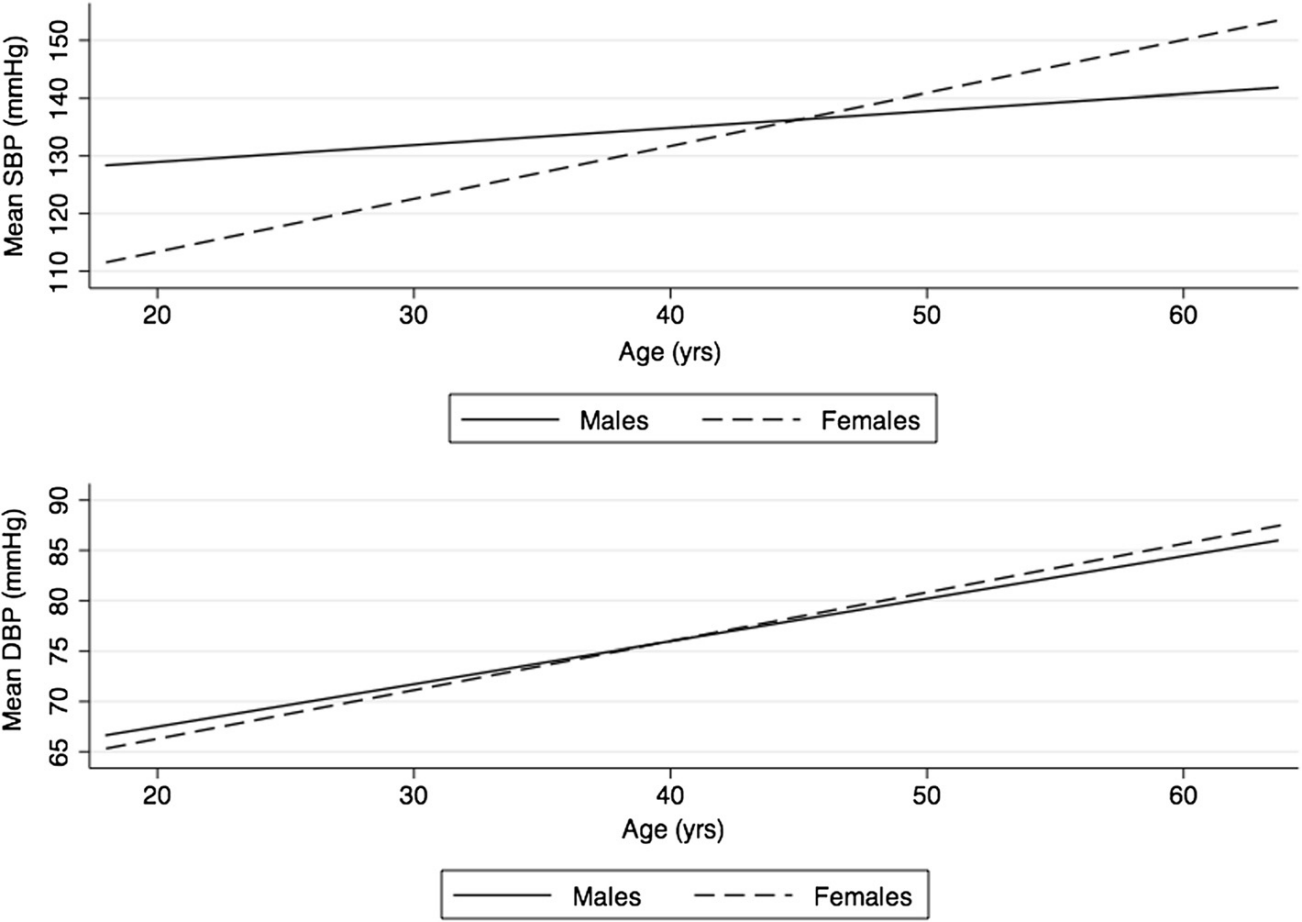
Systolic (SBP) and diastolic blood pressure (DBP) values by sex and age.

The estimated mean SBP and DBP for the population were 129.8 mmHg (95% CI: 128.9 mmHg to 130.7 mmHg) and 73.2 mmHg (95% CI: 72.6 mmHg to 73.9 mmHg) respectively. Both mean SBP and mean DBP increased with age, with a mean SBP of 125.4 mmHg (95% CI: 124.5 mmHg to 126.2 mmHg) and mean DBP of 70.2 mmHg (95% CI: 69.5 mmHg to 70.9 mmHg) in the younger age group compared to a mean SBP of 140.5 mmHg (95% CI: 138.1 mmHg to 142.8 mmHg) and mean DBP of 80.6 mmHg (95% CI: 79.3 mmHg to 81.9 mmHg) in the older age group. A steeper increase of mean SBP (p<0.01) and mean DBP (p=0.03) with age was observed in women in comparison to men (Figure 1).

### Hypertension prevalence, awareness, treatment, control and follow-up

**Figure 2:**
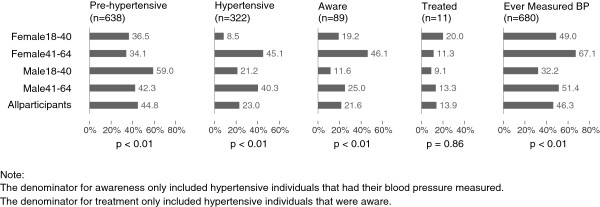
Prevalence of pre-hypertension, hypertension, awareness treatment and “ever measured blood pressure (BP)” per sex-age strata.

The estimated prevalence of pre-hypertension was 44.8% (95% CI: 42.3% to 47.4%) and of hypertension was 23.0% (95% CI: 21% to 25.2%). Figure 2 shows the prevalence of pre-hypertension, hypertension, awareness, treatment and “ever measured BP” per sex-age strata. Men had a higher proportion of hypertension (26.4%; 95% CI: 23.1% to 30.0%) than women (19.8%; 95% CI: 17.5% to 22.3%) (p<0.01) (Table 2). When looking at specific sex-age strata, younger women had the lowest prevalence of hypertension (8.5%; 95% CI: 6.5% to 11.0%) and older women the highest (45.1%; 95% CI: 39.1% to 51.3%) (p<0.01). Amongst hypertensive individuals, 21.6% (95% CI: 17.0% to 26.9%) were aware of their status. This prevalence of awareness was significantly (p<0.01) higher in female subjects (27.5%; 95% CI: 20.3% to 36.1%) compared to male subjects (15.3%; 95% CI: 10.5% to 21.7%) and in the older age group (36.4%; 95% CI: 29.7% to 43.8%) compared to the younger age group (15.4%; 95% CI: 10.1% to 22.8%) (p<0.01) (Table 2). Eighty-three percent of the participants who where aware of their hypertensive status reported having received advice on non-pharmacological management of hypertension, 90% of which were related to salt intake. However just 13.9% (95% CI: 5.9% to 29.1%) (n=11) of the subjects aware of their condition were under pharmacological treatment, of which only 36.4% (n=4) were controlled. Less than half of the participants (46.3%; 95% CI: 43.8% to 48.8%) had ever seen their blood pressure measured. The proportion was higher in older individuals, especially amongst women (67.1%; 95% CI: 61.1% to 72.6%) than in younger individuals, especially men (32.2%; 95% CI: 28.0% to 36.7%) (p<0.01).

**Table 2:** Prevalence of hypertension and awareness according to demographic characteristics, BMI, WHR, alcohol and tobacco use

**Associated factor**	**% Hypertensive (95% CI)**	**P-value**	**% Aware (95% CI)**	**P-value**
**Sex**				
**Female**	19.8 (17.5-22.3)	0.002	27.5 (20.3 - 36.1)	0.01
**Male**	26.4 (23.1-30)		15.3 (10.5 - 21.7)	
**Age**				
**18-40**	14.8 (12.8-17.1)	< 0.0001	15.4 (10.1 - 22.8)	< 0.0001
**41-64**	42.9 (38.1-47.8)		36.4 (29.7 - 43.8)	
**Education**				
**None**	35.3 (30.3-40.6)	< 0.0001	22.3 (14.9 - 31.9)	0.28
**1-4 years**	20.8 (17.0-25.3)		15.5 (8.8 - 25.9)	
**5-8 years**	19.9 (16.6-23.6)		27.6 (18.8 - 38.5)	
≥**9 years**	17.9 (13.5-23.4)		17.3 (8.2 - 32.9)	
**Body Mass Index**				
**<25**	20.1 (17.8-22.7)	< 0.0001	23.5 (17.5 - 30.7)	0.41
**25-30**	28.7 (23.8-34.2)		20.3 (12.9 - 30.5)	
≥**30**	38.1 (29.1-48.0)		14.5 (7.1 - 27.3)	
**Waist to hip ratio**				
**<0.85 women/<0.9 men**	19.0 (16.5-21-8)	< 0.0001	18.7 (12.8-26.4)	0.26
**≥0.85 women/≥0.9 men**	29.0 (25.6-32.7)		24.3 (17.9-32.1)	
**Alcohol consumption**				
**<3 d/wk**	19.4 (16.8-22.3)	< 0.0001	29.3 (21.5-38.5)	0.007
≥**3 d/wk**	26.9 (23.7-30.4)		15.6 (10.8-22.0)	
**Tobacco use**				
**No**	21.4 (19.3-23.6)	< 0.0001	23.8 (18.7-29.9)	0.009
**Yes**	35.6 (28.1-43.9)		8.3 (3.4-18.7)	

Weighted prevalences shown.

CI: Confidence Interval.

**Figure 3:**
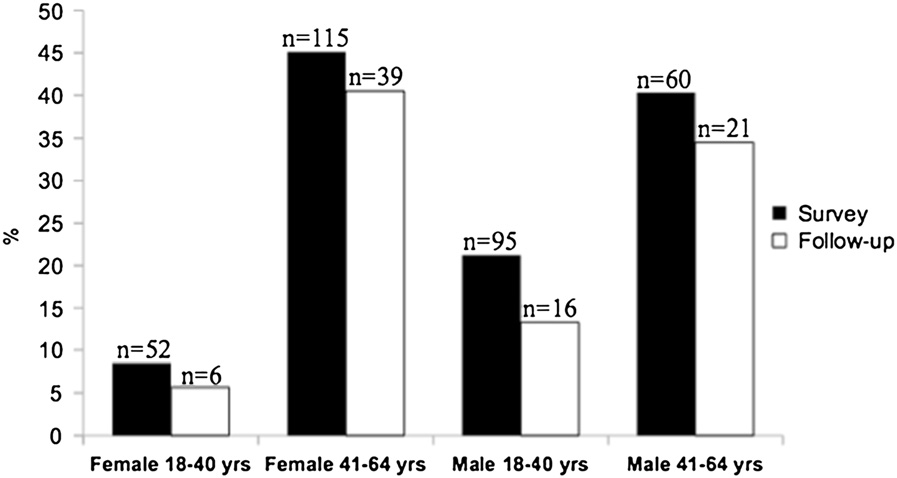
Differences in hypertension prevalence between field-survey and follow-up consultation.

Just over one fourth (27.6%) of hypertensive subjects attended the follow-up consultation at the district hospital. The mean waiting time between field measurements and follow-up was 22.9 days (SD 10). The older age group had a significantly (p<0.01) higher proportion of attendants (34.4%; 95% CI: 27.6% to 41.9%) than the younger (15.3%; 95% CI: 10.3% to 22.2%). 81.7% of follow-up patients had levels of SBP and/or DBP above the defined cut-offs for hypertension diagnosis, therefore confirming their status. Younger participants had a lower proportion (64.2%; 95% CI: 41.9% to 81.7%) of confirmed hypertensive compared to the older age group (87.9%; 95% CI: 76.2% to 94.2%) (p=0.03). Figure 3 compares prevalence levels of hypertension at follow-up to levels at the time of field survey' administration by stratum.

Fifty-five participants (23.0%) who did not attend the follow-up consultation answered the questionnaire on perceived barriers to attending this appointment. Lack of time (41.8%), money (40%) and means of transportation (34.5%), were the three major reasons pointed out by respondents.

### Hypertension associated factors

In general, hypertension was strongly associated with older age (p<0.01) with an OR of 7.2 (95% CI: 4.7 to 10.9; p<0.01) in women and an OR of 1.7 (95% CI: 1.1 to 2.6; p<0.05) in men (Table 3). Younger women had the lowest hypertension prevalence (8.5%; 95% CI: 6.5% to 11.0%), followed by younger men (21.2%; 95% CI: 17.6% to 25.2%), older men (40.3%; 95% CI: 32.7% to 48.3%) and finally older women (45.1%; 95% CI: 39.1% to 51.3%) (Table 3).

**Table 3:** Hypertension associated factors and corresponding multivariate adjusted odds ratios by gender

**Gender**	**Associated factor**		**% Hypertensive (95% CI)**	**Adjusted OR (95% CI)**
**Women (n=866)**				
	**Age**	18-40 (n=611)	8.5 (6.5-11.0)	-
		41-64 (n=255)	45.1 (39.1-51.3)	7.2 (4.7-10.9)‡
	**Education**	≥9 years (n=74)	8.2 (3.8-17.0)	-
		5-8 years (n=246)	10.4 (7.1-14.9)	1.2 (0.4-3.3)
		1-4 years (n=295)	17.3 (13.4-22.0)	1.6 (0.6-4.3)
		None (n=251)	35 (29.5-40.9)	2.6 (1.0-7.2)*
	**Body Mass Index**	<25 (n=567)	17.4 (14.7-20.6)	-
		25-30 (n=214)	22.0 (17.0-28.0)	1.3 (0.8-2.0)
		≥30 (n=85)	29.9 (21.2-40.5)	2.0 (1.1-3.6)†
	**Abdominal obesity**	No (n=369)	15.9 (12.6-19.9)	-
		Yes (n=497)	22.6 (19.3-26.4)	1.1 (0.7-1.6)
	**Alcohol use**	No (n=534)	18.7 (15.8-22.1)	-
		Yes (n=331)	21.7 (17.7-26.3)	1.4 (0.9-2.1)*
	**Tobacco use**	No (n=829)	18.8 (16.5-21.4)	-
		Yes (n=36)	39.1 (24.7-55.7)	0.9 (0.4-2.0)
**Men (n=598)**				
	**Age**	18-40 (n=449)	21.2 (17.6-25.2)	-
		41-64 (n=149)	40.3 (32.7-48.3)	1.7 (1.1-2.6)†
	**Education**	≥9 years (n=169)	21.0 (15.5-27.8)	-
		5-8 years (n=269)	26.2 (21.3-31.7)	1.3 (0.8-2.1)
		1-4 years (n=87)	29.4 (20.7-39.8)	1.4 (0.7-2.6)
		None (n=73)	36.0 (25.8-47.6)	1.8 (0.9-3.4)*
	**Body Mass Index**	<25 (n=495)	22.4 (19.0-26.3)	-
		25-30 (n=85)	40.7 (30.8-51.4)	1.9 (1.1-3.3)†
		≥30 (n=18)	65.7 (41.8-83.6)	4.2 (1.4-13.0)†
	**Abdominal obesity**	No (n=473)	20.8 (17.4-24.7)	-
		Yes (n=125)	46.9 (38.4-55.7)	2.5 (1.5-4.0)‡
	**Alcohol use**	No (n=247)	20.5 (15.9-26.0)	-
		Yes (n=351)	30.5 (26.0-35.5)	1.4 (0.9-2.2)*
	**Tobacco use**	No (n=490)	24.6 (21.0-28.6)	-
		Yes (n=107)	34.7 (26.3-44.2)	1.6 (1.0-2.7)*

Weighted prevalences shown.

*p<.10; †p<.05; ‡p<.01.

95% CI: 95% confidence interval.

OR: odds ratios.

Factor categories with no response from participants may not always equal the total n for a given group.

The prevalence of hypertension increased as the level of education decreased, being 17.9% (95% CI: 13.5 to 23.4) in those with high school or college education, in contrast to 35.3% (95% CI: 30.3% to 40.6%) in individuals with no formal education (p<0.01) (Table 2). Looking at each gender separately, the association between hypertension and having no formal education did not reach statistical significance at the 0.05 level in either women (OR: 2.6; 95% CI: 1.0 to 7.2; p=0.06) or men (OR: 1.8; 95% CI: 0.9 to 3.4; p=0.09). Hypertension was found to be more common in individuals presenting higher values of BMI. The estimated prevalence of hypertension was almost doubled when comparing the non-overweight/non-obese group (20.1%; 95% CI: 17.8 to 22.7) to the obese group (38.1%; 95% CI: 29.1 to 48.0) (p<0.01) (Table 2). This association between hypertension and obesity was significant in both men (OR: 4.2; 95% CI: 1.4 to 13.0; p<0.05) and women (OR: 2.0; 95% CI: 1.1 to 3.6; p<0.05) (Table 3). Among abdominally obese subjects, the estimated prevalence of hypertension was higher (29.0%; 95% CI: 25.6 to 32.7) than in those non-abdominally obese (19.0%; 95% CI: 16.5% to 21.8%) (Table 2). However, the association between hypertension and abdominal obesity was only significant in men (OR: 2.5; 95% CI: 1.5 to 4.0; p<0.01) (Table 3).

Hypertension prevalence was significantly higher in frequent drinkers (26.9%; 95% CI: 23.7% to 30.4%) compared to non-frequent drinkers (19.4%; 95% CI: 16.8% to 22.3%) (p<0.01) and significantly higher in smokers (35.6%; 95% CI: 28.1% to 43.9%) than in non-smokers (21.4%; 95% CI: 19.3% to 23.6%) (p<0.01) (Table 2). Nevertheless, when looking at each gender separately the association between hypertension and alcohol or tobacco use was only marginally significant in men with an OR of 1.4 (95% CI: 0.9 to 2.2; p=0.08) and 1.6 (95% CI: 1.0 to 2.7; p=0.07), respectively (Table 3).

## Discussion

In recent years there has been an effort to provide reliable data regarding hypertension in SSA, predicted to be the leading risk factor for CVDs and accounting for a high burden of morbidity and mortality [1]. To our knowledge this is the first population-based survey to provide insightful data on hypertension prevalence and associated factors in Angola.

One fourth of our DSS population was estimated to be hypertensive and almost half of the individuals presented SBP and DBP values compatible with pre-hypertension. These prevalence levels are within the range of reported values in surveys performed in SSA using the STEPS methodology, being lower than the 41% reported for urban Mozambique [18] but higher than the overall 16% published for Eritrea [19]. Due to the methodological differences namely in sampling frames, age groups and measurement techniques used, direct comparisons between studies are not possible. Nevertheless our estimates are lower than the 29.5% of overall hypertension found in the U.S. National Health and Nutrition Examination Survey (NHANES), 2009 to 2010 cycle, [20] and also lower than sex-specific prevalence rates reported for Europe (ranging from 29.3% in Western Europe to 45.6% in Northern Europe) [6].

Roughly half of the adult population in the DSS study area had never had their blood pressure measured by a healthcare professional and less than one-fourth of the hypertensive subjects were aware of their condition. This value of awareness is lower than the 74.0% found in the U.S, [20] and than the 40.7% in Northern Europe and the 70.8% in Central and Eastern Europe [6]. When compared to other studies performed in SSA our results are higher than the 14,8% found in Mozambique [18] but lower than the 34% in Ghana [21] where a similar study has been performed. As in similar studies, the prevalence of awareness was higher in women, especially in the older age group. Previous authors [18,22] have argued that these findings might be due to the privileged relationship women have with health care facilities because of maternal and child-care oriented programs [6]. Among hypertensive patients aware of their condition, less than 15% (n=11) were under pharmacological treatment, of which only four people fulfilled criteria for control. Due to these low numbers it was not possible to make a comparison of control across the sex and age strata. The lack of awareness, treatment and control might alert for the possibility that, despite being a growing economy, Angola´s health system may not be currently able to provide adequate cardiovascular health care [23]. Furthermore, the extremely low percentage of hypertensive under treatment may highlight the challenge of convincing these patients to take lifelong medication in order to treat a disease, which is usually asymptomatic. In fact, since a frequent reason (41.8%) given by participants for not attending the follow-up consultation was lack of time, it is likely that the hypertensive diagnosis was not considered a priority, suggesting little awareness of the consequences of this condition among our study population. In addition, money and transportation were also stated as important limitations, showing that socio-economic constraints might be an important limitation to health-care seeking behaviours.

The prevalence of hypertension was significantly higher among women of older age when compared to other strata. In fact, the association with older age proved to be stronger in women than in men (OR of 7.2 versus 1.7; p<0.01). The estimated prevalence of overweight in our study was 40%, higher than 11.7% found in Mozambique [18] for comparable age-sex strata, whereas obesity was 15%, also higher than the 5.2% reported for that same country and the double of the value reported for Eritrea [18,19]. Even though obesity was more common among older women, the association with hypertension was stronger in men than in women (OR of 4.2 versus 2.0; p<0.05). In previous studies addressing the relationship of obesity and hypertension this effect has been seen, but no clear explanation was provided for these findings [19]. Due to the low absolute numbers of obese hypertensive in our survey this effect has to be interpreted with caution.

Frequent alcohol consumption (on 3 or more days per week) was reported by more than half the participants of our survey. Although methodological differences in measuring alcohol consumption do not allow for direct comparisons between studies, our estimates are higher than the 16.6% previously reported by Damasceno et al. [18] and than the 18% reported by Mathenge et al. [24]. Alcohol use has been recognized as an important risk factor for overall disease burden in developing countries [2] further justifying the need for future work to better describe this problem in our setting. Another possible limitation of our work is the lack of data regarding other lifestyle risk factors such as unhealthy diet and physical inactivity.

Different studies in SSA, have shown that the prevalence of hypertension is higher amongst groups with a higher level of education in rural settings, whereas the inverse occurs in urban environments [18,25]. Our results did not confirm this hypothesis. Although the prevalence of hypertension was lowest in the highest educational group, this association was not significant after adjusting for gender. Future studies addressing this issue are warranted.

A potential limitation of our study resides on the fact that participants in this survey were mainly from an urban setting. Future studies targeting rural populations are needed to allow for valuable comparisons to studies preformed by others in similar settings [18-22,25].

There is a growing body of evidence showing that hypertension is more prevalent with increasing urbanization, which has lead to sedentarism, less healthy diets, increased alcohol consumption and higher body weights [4,6,18,26,27]. Notwithstanding important socio-economic inequalities still present in the country, Angola's recent massive economic growth is having a profound effect on urban settings, which are becoming more populated and wealthier [10]. In fact, we found a high prevalence of CVDs lifestyle risk factors, which will most likely have an impact on the prevalence of hypertension and associated complications. Our ongoing DSS will allow for future surveys and/or intervention studies to be performed and further enlighten the impact of these behaviors in the prevalence of hypertension and associated CVDs conditions.

Although adequate treatment and follow-up of hypertensive individuals has been shown to prevent CVD, extend and improve quality of life, hypertension remains inappropriately managed worldwide, especially in SSA [4,18,22,28]. In this study, we aimed to encourage treatment and management of this condition among the group of participants identified as hypertensive at the time of field data collection by establishing a follow-up consultation. Just over 80% of the subjects attending this consultation were given a definite hypertension diagnosis, alerting for the fact that the methodology applied might be overestimating hypertension prevalence, as suggested by Bovet et al's work in Tanzania [29]. Therefore future hypertension surveys should have this in consideration as a limitation.

A recent survey on malaria, schistosomiasis, geohelminths, anemia and malnutrition among children and women has shown that these endemic conditions are important public health problems in our setting [12]. Our study, therefore confirms the coexistence of non-communicable diseases with endemic communicable ones in the same area, alerting for the need of innovative public health campaigns to approach this complex epidemiological transition occurring in Angola, in line with previous reports from other SSA countries [18,24]. In view of this, DSSs are uniquely positioned to monitor changes over time and thus characterize socio-economic modifications that go alongside with the epidemiological transition.

## Conclusions

The impact of Angola’s recent fast economic growth on risk factors for CVD, in particular those associated with hypertension, needs to be considered. Future studies assessing additional lifestyle risk factors for hypertension such as unhealthy diet and physical inactivity are warranted to understand the potential impact that this economic boom might have on hypertension prevalence. Data provided here call for the implementation of public health policies to promote primary prevention, early detection and access to effective treatment options for hypertension in Angola.

## Competing interests

The authors declare that they have no competing interests.

## Authors’ contributions

JEP participated in the design of the project, led data collection process in the field, participated in data analysis and drafted the paper. YVS participated in data collection, performed the statistical analysis and revised subsequent drafts of the paper. AJL was responsible for data entry and management. SVN participated in the study design and analysis, coordinated its implementation and revised subsequent drafts of the manuscript. All authors read and approved the final manuscript.

## Pre-publication history

The pre-publication history for this paper can be accessed here:

http://www.biomedcentral.com/1471-2458/13/90/prepub
